# Adult Stroke Screening Tool in Childhood Ischemic Stroke

**DOI:** 10.15844/pedneurbriefs-31-1-3

**Published:** 2017-01

**Authors:** Kathleen M. Gorman, Mark S. Wainwright

**Affiliations:** 1 Ruth D. & Ken M. Davee Pediatric Neurocritical Care Program, Ann & Robert H. Lurie Children’s Hospital of Chicago, Chicago, IL; and Departments of Pediatrics and Neurology, Northwestern University Feinberg School of Medicine, Chicago, IL

**Keywords:** Ischemic Stroke, Pediatric, Stroke Detection, Stroke Recognition, Stroke Scale

## Abstract

Investigators from Nationwide Children's Hospital, Columbus, OH performed a retrospective study to determine whether the application of an adult stroke scale could discriminate between children with acute arterial ischemic stroke (AIS) and other causes of acute neurologic deficits.

Investigators from Nationwide Children's Hospital, Columbus, OH performed a retrospective study to determine whether the application of an adult stroke scale could discriminate between children with acute arterial ischemic stroke (AIS) and other causes of acute neurologic deficits. They used the Central Ohio Trauma System (COTS) scale, a 4-point scale of clinical signs; decreased consciousness, slurred speech, facial droop and unilateral arm drift. [1]

COMMENTARY. The recognition of AIS in children is challenging compared to adults, with an average delay of 22 hours to diagnosis [2]. In adult neurology practice, there are a number of validated tools to assist quicker recognition of AIS and subsequent earlier interventions. The utility of adult stroke tools for identification of pediatric AIS has been examined in small retrospective studies [3]. Neville and Lo found that total COTS scale failed to help distinguish AIS from non-stroke patients [1]. Retrospective analysis of the FAST (Face Arm Speech Test) and ROSIER (Recognition of Stroke in the Emergency Department) scales in the pediatric setting found that both are less sensitive compared to adult patients [3]. These studies reinforce the need to develop pediatric-specific tools.

The aim of implementing a stroke scale is to help discriminate AIS from non-stroke acute neurological deficits based on the clinical signs at presentation. In a previous study of single cohort of children presenting with focal neurological deficits, stroke mimics accounted for 54% of the children screened [4]. The clinical signs and symptoms of AIS can be subtle and harder to identify especially in infants and toddlers due to their development stage. The presence of slurred speech is a fundamental component in adult stroke scales but could only be assessed in 61% of this cohort [1]. In a sub-analysis, the authors concluded that the presence of unilateral arm weakness or seizures in pediatric population was significantly associated with AIS [1]. This suggests arm weakness and seizures should be included as criteria in pediatric stroke scales, however larger studies are needed to verify this observation.

Total COTS score was calculated from pediatric neurologists notes in the AIS population compared to emergency physicians in the cohort group. The diagnosis of AIS was often radiologically confirmed prior to neurology exam, potentially biasing the COTS score. The control group only included focal motor deficits, excluding patients who presented with sensory deficits, acute vision loss, aphasia or focal seizures. In previous studies, stroke code was triggered by multiple neurological presentations including seizures and falls [5]. This study omitted a large group of patients who were screened and potentially treated by stroke teams in other institutions. The establishment of a multidisciplinary acute stroke code service (Neurology, ED, PICU, anesthetics, radiology, pharmacy) requires significant investment, training and resources to enable immediate response to AIS and potential thrombolytic treatment [5]. This study did not address the question of whether COTS allowed earlier recognition of AIS and implementation of the stroke code.

This study adds to the data which show that adult stroke scales do not have the sensitivity or specificity to differentiate AIS from other causes of acute focal deficits in children. Pediatric stroke scales need to be developed with the awareness of the limitations of pediatric examination, variability in clinical presentation and the developmental age of the child. The findings from this study suggest that unilateral arm weakness may be an important component of any proposed pediatric-specific scale.
